# Etiology and Viral Genotype in Patients with End-Stage Liver Diseases admitted to a Hepatology Unit in Colombia

**DOI:** 10.1155/2011/363205

**Published:** 2011-09-20

**Authors:** Fabian Cortes-Mancera, Carmen Luisa Loureiro, Sergio Hoyos, Juan-Carlos Restrepo, Gonzalo Correa, Sergio Jaramillo, Helene Norder, Flor Helene Pujol, Maria-Cristina Navas

**Affiliations:** ^1^Grupo de Gastrohepatología, Facultad de Medicina, Universidad de Antioquia, Medellín, Colombia; ^2^Instituto Tecnológico Metropolitano (ITM), Institución Universitaria Adscrita a la Alcaldía de Medellín, Medellín 549 59, Colombia; ^3^Laboratorio de Virología Molecular, CMBC, Instituto Venezolano de Investigaciones Científicas, Apdo. 20632, Caracas 1020A, Venezuela, Caracas, Venezuela; ^4^Unidad de Hepatología y Trasplante Hepático, Hospital Pablo Tobón Uribe (HPTU), Calle 78B 69-240, Medellín, Colombia; ^5^Department of Virology, Swedish Institute for Infectious Disease Control, 171 82 Solna, Sweden

## Abstract

Hepatitis B virus (HBV) and hepatitis C virus (HCV) infections are the principal risk factor associated to end-stage liver diseases in the world. A study was carried out on end-stage liver disease cases admitted to an important hepatology unit in Medellin, the second largest city in Colombia. From 131 patients recruited in this prospective study, 71% of cases were diagnosed as cirrhosis, 12.2% as HCC, and 16.8% as cirrhosis and HCC. Regarding the risk factors of these patients, alcohol consumption was the most frequent (37.4%), followed by viral etiology (17.6%). Blood and/or hepatic tissue samples from patients with serological markers for HCV or HBV infection were characterized; on the basis of the phylogenetic analysis of HCV 5′ UTR and HBV S gene, isolates belonged to HCV/1 and HBV/F3, respectively. These results confirm the presence of strains associated with poor clinical outcome, in patients with liver disease in Colombia; additionally, HBV basal core promoter double mutant was identified in HCC cases. Here we show the first study of cirrhosis and/or HCC in Colombian and HBV and HCV molecular characterization of these patients. Viral aetiology was not the main risk factor in this cohort but alcohol consumption.

## 1. Introduction

Hepatitis B virus (HBV) and hepatitis C virus (HCV) infections are a serious health concern due to their global distribution and direct relationship with liver cirrhosis and hepatocellular carcinoma (HCC) development. In average, 57 and 78% of cirrhosis and HCC cases are attributable to these hepatotropic viruses [1, 2]. HCC is the most common primary liver tumor that presents a heterogeneous prevalence among different ethnic groups and geographic regions. More than 80% of HCC cases occur in Asia and Africa: Japan, China, and Niger, holding an incidence of 20–500 cases/100.000 inhabitants; South American countries present a lower incidence (5 cases/100.000 inhabitants), particularly in Colombia; an incidence of 2 cases/100.000 inhabitants is estimated [3].

Although clinical significance of the HBV and HCV genotypes has not been completely elucidated, increasing epidemiological data suggests that some genotypes could be related to higher risk of HCC development [4–8]. For example, the HCV subtype 1b (HCV/1b) is associated with more severe clinical outcome and poor antiviral response. Moreover patients infected with HBV genotype C (HBV/C) present a higher frequency of HCC than HBV/B-infected patients [9–13]; patients with HBV/F seem to follow a similar tendency like HBV/C-infected patients [14, 15]. In the same way, a double mutation (A1762T/G1764A) in HBV basal core promoter (BCP) region has been implicated with severe clinical outcome and poor response to nucleosides' analogues [4, 16–20].

Considering that Latin American region is extremely diverse in culture, ethnicity, socioeconomic status, and health systems, the research findings of neighbour countries are not totally comparable. Until now, the epidemiological pattern of end-stage liver diseases in Colombia is unknown, and no report has described the molecular characterization of HBV and HCV in this group of patients [21, 22]. In the present study, aetiology and viral genotypes, subgenotypes/subtypes, and HBV pre-C/C mutants were analyzed in cirrhosis and/or HCC cases attended at the Pablo Tobon Uribe Hospital (HPTU) during the period 2005–2007 in Medellin, the second largest city in Colombia.

## 2. Materials and Methods

### 2.1. Patients

From February 2005 to February 2007, 131 patients with end-stage liver diseases (cirrhosis and/or HCC) were enrolled in this study; previous voluntary informed consent sign was obtained. All patients were recruited at the Hepatology Unit of HPTU in Medellin city, Colombia. Diagnosis of liver cirrhosis was established according to the following findings: hepatic encephalopathy, ascites, digestive bleeding due to esophageal varices, coagulopathy, spontaneous bacterial peritonitis, hepatorenal syndrome, imaging criteria (ultrasonography, magnetic resonance, and tomography), and/or liver biopsy; HCC diagnosis was performed following the guidelines of the European Association for the Study of the Liver (EASL).

### 2.2. Samples

Serum and liver tissue samples were obtained from patients who underwent liver transplantation. Both types of material were kept at −70°C until processing; the following serological markers were assessed in samples: HBsAg, total anti-HBc, and anti-HCV (Roche).

### 2.3. Viral Genome Detection

Total RNA and DNA were isolated from samples with serological markers for HBV and/or HCV, using Trizol reagent (Invitrogen, USA). HBV DNA was amplified by PCR, using S-gene-specific primers [23]. The HCV genome was assessed by nested RT-PCR, using flanking primers for 5′UTR, following a protocol previously published [24, 25]. As a positive control, samples with HBV or HCV genome detection were used; liver tissue from a patient with diagnosis of cirrhosis associated to alcohol intake abuse, without HBV or HCV infection, was used as negative controls. All assays were performed in duplicate.

### 2.4. Molecular Characterization of HBV and HCV

In order to know the viral genotype, different PCR products were sequenced to perform a phylogenetic analysis; HCV genotyping was conducted with the 5′ conserved region (5′UTR), using the primers' set described before for HCV detection [24, 25]. To amplify the full HBV genome (3200 nts), a first round of PCR was performed with P1 and P2 primers [26]. A second round of PCR was carried out with some isolates using primers 58p-1450n, 1860p-2853n, 2440p-58n, 1101p-P2, P1-2440n, and 1450p-P2, to obtain the total genome sequences by subregions' amplification [27]. When total HBV genome was not amplified with the primers mentioned above, the small S gene fragment was amplified using 58P-1101N in the first round and s3-s3as (319 nt) in a second round [27, 28], or hep3-hep33 as a unique round of PCR [29].

All sequences obtained were compared with GenBank-available sequences of known genotypes, including subgenotypes/subtypes. Phylogenetic analyses by neighbour joining, maximum parsimony, and maximum likelihood were conducted with PAUP 4.0, MEGA 4.1; Treeview program was used for tree representation. Recombination events were studied by bootscanning and similarity analysis (Simplot).

To evaluate mutations in HBV BCP (A1762T/G1764A) and pre-C/C (G1896A), analysis of the sequences was carried out by comparison with other GenBank sequences of different HBV genotypes considering mutants and wild type. BioEdit program was used for this purpose. The accession numbers of sequences included are as follows: HBV: FJ589065; FJ589066; FJ589067; FJ589068; FJ589069; FJ589070; HCV: JF693486, JF693487, JF693488, JF693489.

## 3. Results

### 3.1. Demographic and Clinical Characteristics of Patients

The mean age of the 131 individuals was 58.1 years (range: 17–85 years); most of patients recruited were males (65.6%). According to the followed clinical guidelines, 71% evidenced cirrhosis, 12.2% HCC, and 16.8% cirrhosis and HCC (HCC/Ci*). Interestingly, when risk factors were analyzed alcohol intake abuse was the most frequent risk factor (37.4%), followed by viral etiology (17.6%), autoimmunity (9.9), NASH (7.6%), and other causes such as metabolic disorders and biliary disease (16.8%); 10.7% of the cases were not associated to any risk factor assessed (Table 1). The most frequent clinical manifestations were esophageal varices (64%), ascites (61.8%), coagulopathy (46%), and hepatic encephalopathy (38.2%); most of patients were scored at the Child B and C (>75%), indicating an advanced chronic liver disease in patients enrolled.

From 131 patients included in the present study, 14 were positive for the HBsAg serological marker (10.7%) and 9 for anti-HCV (6.9%); most of patients infected by HBV and HCV were males (60.8%). The mean age of these 23 patients was 56.6 (range 34–74 years). Among them, 22 had diagnosis of liver cirrhosis and 7 had HCC in addition (HCC/Ci*); just one patient had diagnosis of HCC without cirrhosis. According to the phenotype, 20 patients corresponded to non-Amerindian individuals; besides, the three others were patients from El Salvador, Venezuela, and Israel (Table 2). 

### 3.2. Phylogenetic Analysis

**Figure 1:**
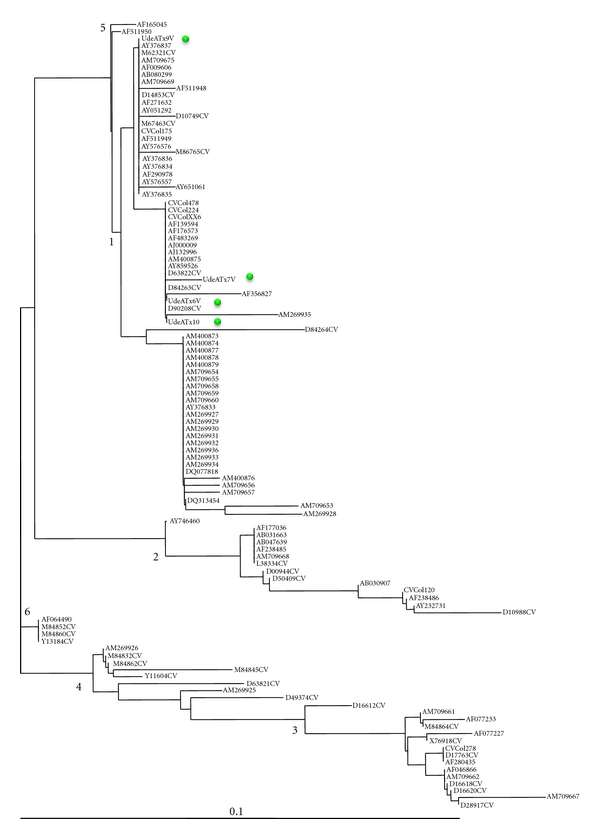
Unrooted Tree generated with MEGA software, using 5'UTR HCV sequences. Solid green circle indicates the position of isolate characterized in the present study. The accession number of the sequences are shown.

In 4 out of 8 tissue samples from patients infected by HCV, it was possible to successfully sequence the 5′UTR. The expected grouping was observed among Genbank sequences after phylogenetic analysis conduction, with minor method-dependent changes (data not shown). All four isolates belonged to HCV genotype 1. Three HCV strains corresponded to HCV subtype 1b and one to subtype 1a (Figure 1).

**Figure 2:**
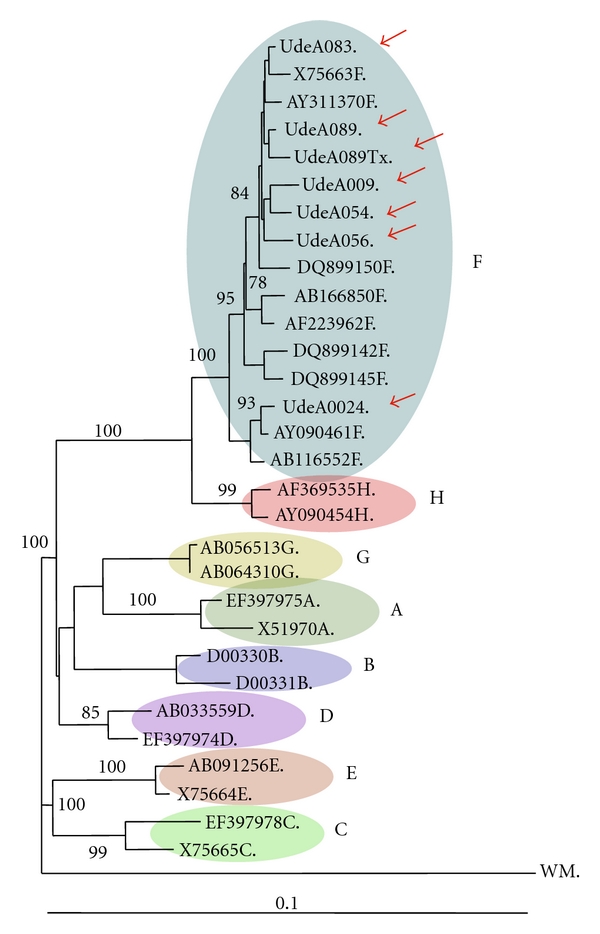
Phylogenetic tree of HBV genotype A to H generated by the Neighbour Joining method (PAUP), using HBV S gene sequences The Hepatitis Woolly monkey virus (WM) sequence was used as outgroup. Red arrow: isolate characterized from cirrhosis and/or HCC cases. The accession number followed by genotype identity is indicated. Bootstrap values are shown (1000 repetitions). HKY was used to assess distances.

In the case of HBV, seven strains were sequenced; four strains from liver tissues and three were serum sample derived. In one additional isolate (UdeA-072) the HBV genome was detected by PCR, although it was not possible to obtain a clear electropherogram after several assays. HBV S gene sequence analysis showed that all isolates belonged to genotype HBV/F, validated by the HBV GenBank sequence grouping results and high bootstrap values observed in most tree branches. Similar topology was observed among trees generated by the different inference methods (data not shown). Five isolates grouped into the clade of South American strains (Codes UdeA-009, UdeA-054, UdeA-056, UdeA-083, and UdeA-089); this clade included the first Colombian isolate characterized by Norder et al. [29]. Interestingly, one of the sequences analyzed (Code UdeA-024) was less related to this clade (Figure 2).

**Figure 3:**
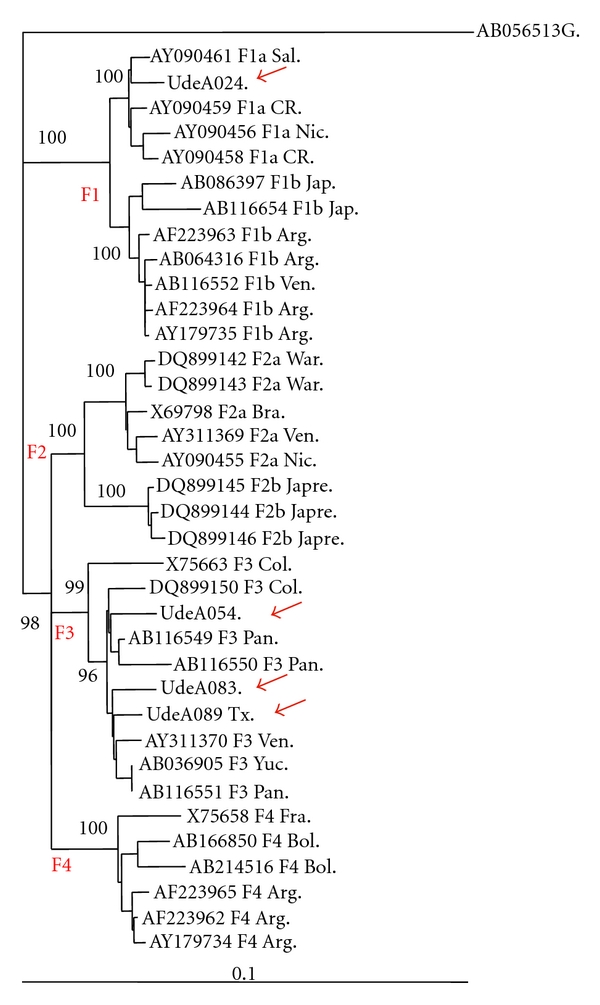
HBV subgenotyping (F1-F4), based on the complete genome analysis using PAUP program. Sequence of genotype G was used as outgroup (AB056513). The accession number and subgenotype are indicated followed by the isolate origin letters code (Sal: El Salvador, CR: Costa Rica, Nic: Nicaragua, Jap: Japan, Arg: Argentina, Ven: Venezuela, Col: Colombia, Pan: Panama, Bol: Bolivia, and two Amerindian tribes from Venezuela War: Warao tribe, Japre: Japreira tribe). Red arrow: sequences belonging to the present study. Bootstrap values are shown (1000 replications).

The complete HBV genome was sequenced in four strains, two Colombian isolates (Codes UdeA-083 and UdeA-089) one from Venezuela (Code UdeA-054), corresponding to subgenotype F3 (HBV/F3), and the sequence from El Salvador (Code UdeA-024), grouped in a different clade from the HBV/F3 (Figure 3). Indeed, UdeA-024, belonging to subgenotype F1a (HBV/F1a), was more closely related to strains from Central America countries (El Salvador, Costa Rica, and Nicaragua), in agreement with the origin of the patient. In the same phylogenetic analysis, partial sequences (S gen) of strains UdeA-009 and UdeA-056 were added. On the basis of strict consensus tree generated by maximum parsimony, these isolates corresponded to HBV/F3; this grouping was supported by a bootstrap value higher than 80. 

The results obtained in the present phylogenetic analysis are in agreement with the HBV and HCV genotype geographic distribution in Latin America. Additionally, this report corresponds to the first description of HBV/F in Colombian patients with severe liver disease.

### 3.3. Characterization of G1896A and A1762T/G1764A Mutants

To establish the presence of G1896A and A1762T/G1764A mutants, pre-C/C sequences were aligned with HBV wild-type and mutant prototypes available in GenBank. The BCP analysis showed that isolates UdeA-083 and UdeA-089 carried the double mutation A1762T/G1764A. These isolates were recovered from the Colombian patients with diagnosis of HCC/Ci* (Table 3). In addition, T at 1858 nucleotide (T^1858^) was detected in isolates UdeA-024 and UdeA-054 and C^1858^ in samples UdeA-083 and UdeA-089. In UdeA-054, mutant G1896A was also identified in addition to T^1858^. The G1896A mutation correlated with detection of no HBeAg by ELISA in the corresponding serum sample (Table 3).

## 4. Discussion

This paper corresponds to the first study of aetiology description in Colombian patients with end-stage liver diseases and the molecular characterization of HBV and HCV strains detected in this group of patients. 

One of forty deaths around the world is due to end-stage liver disease. In the present study, 71% of the patients correspond to cirrhosis cases and 29% to HCC, a similar result to other descriptions reported in countries of the region [1, 30, 31]. Among these 131 patients, alcohol intake abuse was the most frequent risk factor observed (37.4%), followed by viral infections (17.6%). Although this epidemiological pattern is usually found in developed countries, the high proportion of males (in general males have a higher alcohol intake than females) in the present study and the HBV vaccination status in Colombia could be contributing to the risk factors' pattern of the population study [32]. On the other hand, the prevalence of cryptogenic cirrhosis and autoimmune liver disease is according to previous reports [1, 30].

As previously mentioned, the frequency of cirrhosis and HCC cases associated to viral etiology in the present study was low (17.6%; 23/131). Only 10.7% (14/131) and 6.9% (9/131) of these patients were positive for serological markers of HBV (HBsAg) or HCV (anti-HCV) infection, respectively.

The HCV-related HCC has increased in several countries; 80% of infected patients with HCV progress to chronic infection, while 20% of them develop cirrhosis, and at least 5% of these evolve to HCC [33]. In Latin America, the world health organization (WHO) estimates an intermediate prevalence of HCV infection (1–2.5%); moreover, a low prevalence (0.5–1%) has been reported among Colombian blood donor population [34–36].

As previously mentioned, Medellin is the second largest city in the country and the capital of Antioquia State (Department of Antioquia). Health authorities in Antioquia have reported a similar HCV prevalence since 2004 (0.2–0.3/100.000 inhabitants) (Indicadores Básicos 2004–2007).

Contrary to general population, studies in some Latin American countries show a high HCV prevalence in severe liver disease. Indeed, HCV infection is the predominant HCC risk factor in Argentina, Chile, and southeastern states of Brazil. Furthermore, in a recently prospective multicenter study of HCC cases from 9 Latin American countries, the main HCC risk factor was HCV infection (30.8%), followed by alcohol (20.4%), HBV infection (10.8%), and then HCV plus alcohol (5.8%) [37–39]. A similar tendency was observed in Mexican cirrhotic patients, where 39.5% of recruited patients presented alcohol intake abuse, followed by HCV infection (36.6%) [40]. 

Considering the analysis of HCV sequences included in the phylogenetic analysis, the prototypes clustered according to the genotypes described in the literature (HCV/1-6), obtaining similar results with all methods conducted. Inside the main cluster of genotype HCV/1 was clearly observed clades assigned to subtype HCV/1a and HCV/1b; Colombian strains were grouping into these subtypes. Indeed, one isolate belongs to HCV/1a and three strains to HCV/1b. This result is consistent with previous reports of HCV geographic distribution in Latin American countries where different genotypes are present (HCV/1, HCV/2, HCV/3, and HCV/4); however, genotype HCV/1 is the prevalent in most countries of the region including Colombia [41]. Indeed, HCV/1 has been described in some studies performed by different approaches in Colombian multitransfused patients, individuals with elevated aminotransferases, general population, and kidney transplant patients [42–45]. This is the first report based on sequence analysis and developed in samples of patients with severe liver disease in Colombia. Secondly, most of research findings agree that HCV/1b is related with higher risk of severe liver disease [23, 46–48]. It is important for health authorities in Colombia and other Latin American countries to develop studies that contribute to knowing the impact of genotype HCV/1b over the hepatitis C natural history in the region.

According to WHO, Colombia has a moderate endemicity for hepatitis B, although there are several epidemiological patterns given the geographic, ethnic, cultural, and socioeconomic status of the population. Actually, Sierra Nevada de Santa Marta, Orinoquian and Amazon basins, and southeastern part of the country corresponded to high-prevalence regions for hepatitis B infection in this country. However Antioquia state holds a different behavior [49]; in fact, the general incidence of HBV infection in this state in the last years range was 2.7–4.4 per 100.000 inhabitants, while the prevalence in blood donors was 0.3% [49, 50]. As mentioned above, HBV infection was observed in 10.7% of the patients analyzed. The study population was recruited in Medellin, the second largest city in Colombia, in one of the most important units of hepatology in the country; even if it is possible that this hospital receives patients from rural area of Antioquia state and other Colombian states, most of the cases corresponded to people living in urban area and not from high-prevalence regions of hepatitis B infection. 

This heterogeneity of hepatitis B situation is also described in Brazil; indeed, higher frequency of HBV infection than other risk factors has been described in HCC patients from states of northeastern and northern regions of Brazil but not in patients from southeastern states [51, 52].

The low HBV prevalence described in the present study contrasts with some studies conducted in Peru and Brazil, where HBV was reported in 42–63% of end-stage liver disease cases [51–56]. Contrary to these reports, a low HBV prevalence has been described among HCC patients from Chile (6.8%) and Puerto Rico (4%) [57, 58], similar to other works carried out in the United States, Japan, and western Europe [1]. On the other hand, on Ecuador a study conducted in 770 cirrhotic patients linked viral etiology to 2.8% of the cases, while alcohol intake was the most frequent risk factor associated (48.3%) [59]. Whereas differences among studied populations (gender, age, origin), diagnosis, and viral markers are described above, additional studies will be necessary for clarifying the real statement of hepatotropic viruses in cases of cirrhosis and HCC/Ci* in Colombia and the region. 

On the other hand, the phylogenetic analysis of HBV showed that HBV/F and subgenotype HBV/F3 were presented in serum and tissue samples of the Colombian population analyzed; subgenotypes HBV/F1a and HBV/F3 were also detected in two cases from El Salvador and Venezuela, respectively. These results are consistent with previously published reports about molecular diversity of these hepatotropic viruses, geographic distribution in Latin America, and their prevalence in severe forms of hepatic disease. In addition, the A1762T/G1764A double mutant, associated according to some authors with a poor clinical outcome, was described in strains isolated from Colombian patients with diagnosis of HCC/Ci*. As mentioned before all isolates of HBV sequenced belonged to HBV/F. It has been proposed that HBV/F is autochthonous to America due to its predominance in different ethnic groups, in particular Amerindian [60]. In Colombia, few studies about HBV molecular characterization have been published. Two of them included samples from blood donor populations, showing a predominance of genotype HBV/F (77–87.23%) [21, 22]. This result is in agreement with our study and previous findings of genetic population founder carried out in the state of Antioquia [61, 62], which revealed that 90% of the genetic pool (mitochondrial DNA) corresponded to Amerindian origin. More recently, in Colombia was detected the genotype HBV/E in nine pregnant women, being the first description of an exclusively African HBV genotype circulating in South America [63]; this result also coincides with high frequency of African haplotypes in population from Choco state, at the pacific coast of Colombia [64]. The subgenotypes HBV/F1-F4 have a specific geographic distribution in America. Indeed, subgenotype HBV/F1a is predominant in Alaska, Nicaragua, Costa Rica, and El Salvador, while subgenotype HBV/F1b in Peru and Argentina. Subgenotype HBV/F2 is prevalent in Venezuela and Brazil and subgenotype HBV/F3 in Panama, Venezuela, and Colombia. Finally, subgenotype HBV/F4 is present in Bolivia and Argentina [20, 65]. Devesa et al. and Alvarado et al. have characterized the HBV subgenotypes in samples from Colombian blood donor isolates; most of the isolates corresponded to HBV/F3. Genotype HBV/F was also recently characterized in samples from blood donor population from Medellin by our group (unpublished data). In the present study, the complete genome analysis in 4 out of 6 HBV isolates from patients with cirrhosis or HCC/Ci* made it possible to classify 3 of them into subgenotype HBV/F3, and one strain into subgenotype HBV/F1a, while partial analysis (small S gene sequence) of two others showed grouping with HBV/F3 prototypes.

The BCP and pre-C/C mutations have been associated with clinical outcome severity. One frequent mutation corresponds to G1896A in the pre-C/C region which leads to a premature stop codon preventing HBeAg synthesis [4, 8, 20, 66]. When this region was analyzed, the G1896A mutation was characterized only in one isolate (UdeA-054), presenting in addition T^1858^, while the isolates UdeA-083 and UdeA-089 carry C^1858^. According to several studies, G1896A is frequent in genotype HBV/F isolates that carry T^1858^. A hypothesis for this coevolution pattern is that hydrogen binding between nucleotides 1858–1896 is necessary to maintain the low stem secondary structure of *ε* signal [67]. The presence of G1896A in isolate UdeA-054 correlated with no detection of HBeAg by ELISA in serum sample (Table 3).

When BCP was analyzed, it was demonstrated that isolates UdeA-083 and UdeA-089 carried the double mutation A1762T/G1764A; these isolates corresponded to two Colombian patients with diagnosis of HCC/Ci*. 

Regarding the prevalence of double mutation in BCP in isolates of genotype HBV/F, there are different results. In fact, in Brazilian patients with chronic infection, the BCP double mutation was described in 90% of HBV/F isolates; this double mutation was not identified in any other samples of these studies [68, 69].

Several authors have proposed that HBV genotype and BCP mutants could be related with liver disease severity. Although HBV/B and HBV/C circulate in Asia, patients with diagnosis of HBV-related HCC present a higher prevalence of HBV/C infection [9, 12, 13]. Similar findings have been reported for HBV/F, in a prospective study of 258 patients with chronic HBV infection; after a mean followup of 94 months, the mortality rate related to liver disease was more frequent in cases of genotype HBV/F than HBV/ A and HBV/D infection [14]. Livingston et al. also described an association of HBV/F and liver disease severity, in particular HCC risk. They compared the frequency of HBV/F in Alaska natives with chronic hepatitis B infection with or without HCC; the frequency of genotype HBV/F was 68% and 18%, respectively [15]. This finding suggested a higher risk of HCC development in HBV/F cases [15, 60]. Sanchez-Tapias et al. and Livingston et al. described that genotype HBV/F, autochthonous to America, could be related with poor clinical outcome and higher HCC risk development; however, a higher number of studies should be developed for a stronger support of these findings. 

In addition, the present study is the first report of A1762T/G1764A in Colombian HBV isolates. Recent studies assign a more important role to A1762T/G1764A in hepatocarcinogenesis than the HBV genotype itself. It has been demonstrated that the BCP double mutation generates a new binding site for the transcription factor HNF1, regulating pgRNA transcription and promoting an enhancement of HBV replicative activity [70]. In patients with HCC due to HBV infection, isolates belonging to genotype HBV/C carry a higher frequency of A1762T/G1764A compared to HBV/B strains [4, 9, 71, 72]. In our study, the presence of BCP double mutation correlates with HCC diagnosis in those patients.

This study corresponds to the first description of end-stage liver diseases and the molecular characterization of HBV and HCV in cirrhosis and HCC/Ci* cases in Colombia. Genotype HCV/1 and genotype HBV/F (subgenotype F3) were detected in samples belonging to Colombian patients. This result agrees with previous studies and in the case of HBV with genetic founder populations in Colombia. Additionally, HBV/F3 and HBV/F1a were characterized in isolates from patients from Venezuela and El Salvador, respectively. The HBV and HCV subgenotyping/subtyping results obtained in the present study are according to the geographic pattern and predominance described for these hepatotropic viruses, especially subgenotypes HBV/F1a and HBV/F3 in Central and South America, respectively.

On the other hand, mutation of A1762T/G1764A was characterized in isolates from patients with HCC. Although the double mutant has been related with higher risk of HCC development, the descriptive design of our study and limited sample size do not allow us to assess any type of statistical association between BCP mutant, genotype, and clinical outcome. Additionally studies will be necessary to determine whether HCV/1b, HBV/F, and pre-C/C variants are associated with a higher risk of cirrhosis and HCC development.

Moreover, the statement of viral etiology and alcohol intake abuse in end-stage liver disease cases in Colombia and Latin America should be explored in further case-control studies.

## Figures and Tables

**Table 1:** Description of End-stage liver disease cases recruited.

Characteristics	Proportion (%)
Risk factors	
Alcohol intake	37,4
Viral etiology	17,6
Cryptogenic	10,7
Autoimmunity	9,9
NASH	7,6
Others*	16,8
Clinical findings	

Esophageal varices	64
Ascites	61,8
Coagulopathy	46
Hepatic encephalopathy	38,2
Spontaneous bacterial peritonitis	15,7
Hepatorenal syndrome	8,9

*Metabolic disorders, biliary disease, or both.

**Table 2:** Clinical and demographic characteristics of patients with positive serological markers for HBV and HCV.

Code	Diagnosis	Origin	Age	Gender	Alcohol*	Type of simple		Serological marker	
						Serum	Liver tissue	HBsAg	Anti-HCV
UdeA-001	Ci	Colombia	68	F	4	NA	Yes	Neg	Pos
UdeA-002	Ci	Colombia	59	M	4	NA	Yes	Neg	Pos
UdeA-003	Ci	Colombia	68	M	4	NA	Yes	Neg	Pos
UdeA-004	Ci	Colombia	47	M	4	Yes	NA	Pos	Neg
UdeA-006	HCC + Ci	Colombia	48	M	4	NA	Yes	Neg	Pos
UdeA-009	Ci	Colombia	69	M	1	Yes	NA	Pos	Neg
UdeA-015	HCC + Ci	Colombia	68	F	9	NA	Yes	Neg	Pos
UdeA-024	Ci	El Salvador	60	M	9	NA	Yes	Pos	Neg
UdeA-054	Ci	Venezuela	47	M	3	Yes	Yes	Pos	Neg
UdeA-056	HCC + Ci	Colombia	56	F	4	Yes	NA	Pos	Neg
UdeA-058	HCC + Ci	Colombia	53	M	9	Yes	NA	Pos	Neg
UdeA-061	Ci	Colombia	47	F	4	Yes	NA	Pos	Neg
UdeA-065	Ci	Colombia	58	F	1	NA	Yes	Neg	Pos
UdeA-069	HCC	Colombia	64	F	4	NA	Yes	Neg	Pos
UdeA-070	Ci	Colombia	34	M	4	NA	Yes	Neg	Pos
UdeA-072	Ci	Israel	49	M	3	Yes	Yes	Pos	Neg
UdeA-077	Ci	Colombia	57	M	1	Yes	NA	Pos	Neg
UdeA-083	HCC + Ci	Colombia	67	F	4	Yes	Yes	Pos	Neg
UdeA-087	Ci	Colombia	48	M	2	Yes	NA	Pos	Neg
UdeA-089	HCC + Ci	Colombia	47	F	4	Yes	Yes	Pos	Neg
UdeA-099	Ci	Colombia	56	M	4	Yes	NA	Pos	Neg
UdeA-101	Ci	Colombia	74	F	4	Yes	NA	Pos	Neg
UdeA-124	HCC + Ci	Colombia	57	M	2	Yes	NA	Pos	Neg

ci: Cirrhosis, HCC: hepatocellular carcinoma, M: male, F: female, *Alcohol intake: for male/(female) 1: >80 g/day (40g/day); 2: 50–80 g/day (20–40 g/day); 3: <50 g/day (20 g/day) 4: no intake; 9: no data; Yes: available; NA: nonavailable; Yes: HBV/HCV-positive sample by molecular analysis; Pos: positive serological result;Neg: negative serological result.

**Table 3:** Molecular characterization of HBV isolates corresponding to End-stage liver disease cases: Genotype, Subgenotype and precore/core mutants.

Code	Diagnosis	Genotype	Subgenotype	Mutation				HBV serological markers		
				pre-C		BCP		HBsAg	IgG anti-HBc	HBeAg
				1858	1896	1762	1764			

UdeA-009	Ci	F	F3^*μ*^	—	—	—	—	Pos	Pos	Neg
UdeA-024	Ci	F	F1a^∞^	T	G	A	G	Pos	Pos	Neg
UdeA-054	Ci	F	F3^∞^	T	A^*α*^	A	G	Pos	Pos	Neg
UdeA-056	HCC/Ci*	F	F3^*μ*^	—	—	—	—	Pos	Pos	Neg
UdeA-083	HCC/Ci*	F	F3^∞^	C	G	T*	A*	Pos	Pos	Neg
UdeA-089^*β*^	HCC/Ci*	F	F3^∞^	C	G	T*	A*	Pos	Pos	Neg

*β*: both, tissue and serum samples, Ci: cirrhosis, HCC/Ci*: cirrhosis and hepatocellular carcinoma, ∞: based on complete genome analysis, *μ*: based on S gene sequence analysis, —: no data, *α*: nonsense mutation, *: double mutant, Pos: positive, Neg: negative. Strains isolated from Colombian patients: UdeA-009, UdeA-024, UdeA-056, UdeA-083, and UdeA-089. Strain isolated from a Venezuelan patient: UdeA-54. 1.
